# Evaluating the Operational Features of an Unconventional Dual-Bay U-Turn Design for Intersections

**DOI:** 10.1371/journal.pone.0158914

**Published:** 2016-07-28

**Authors:** Yun Xiang, Zhibin Li, Wei Wang, Jingxu Chen, Hao Wang, Ye Li

**Affiliations:** 1 Jiangsu Key Laboratory of Urban ITS, Southeast University, Si Pai Lou #2, Nanjing, China; 2 Jiangsu Province Collaborative Innovation Center of Modern Urban Traffic Technologies, Si Pai Lou #2, Nanjing, China; 3 School of Civil Engineering and Architecture, Nanchang Hangkong University, Feng He Nan #696, Nanchang, China; Beihang University, CHINA

## Abstract

Median U-turn intersection treatment (MUTIT) has been considered an alternative measure to reduce congestion and traffic conflict at intersection areas. The MUTIT is sometimes difficult to implement in the field because it requires wide median on arterials for U-turn vehicles. The objective of this study is to introduce an unconventional U-turn treatment (UUT) for intersections which requires less median space but is also effective. The UUT has a dual-bay design with different turning radiuses for small and large vehicles. The VISSIM simulation model was developed to evaluate the operational features of the UUT. The model was calibrated using data collected from intersections in China. The capacity, delay and number of stops were evaluated and compared with the direct-left-turn (DLT) for the same intersections. The results showed that the UUT significantly improved the operations at intersection areas, especially when volume/capacity ratio is small, and ratio of left-turn to through traffic is small. With the UUT, the capacity is increased by 9.81% to 10.38%, vehicle delay is decreased by 18.5% to 40.1%, and number of stops is decreased by 23.19% to 36.62%, when volume/capacity ratio is less than 0.50. The study also found that traffic efficiency could be further improved when the UUT is designed in conjunction with signal control. In the case, the UUT plus signalized control increases the capacity by 25% to 26.02%, decreases vehicle delay by 50.5% to 55.8%, and reduces number of stops by 69.5%, compared with the traditional DLT.

## Introduction

Signal design for left-turn traffic at intersection has been long considered as a dilemma. Providing protected left-turn signal for left-turn vehicles could increase delay to through traffic [1, 2]. Non-protected left-turn signal control could increase conflicts between left-turn vehicles and through traffic on the opposite direction [3, 4]. To reduce such problem, many alternative measures have been proposed to improve the performance of intersections with heavy left-turn traffic, such as the signal timing optimization [5, 6], the exclusive left-turn lane design [7, 8], and some novel techniques such as autonomous vehicle [9, 10]. Among those measures, the facilities design is still an important way to solve existing problems.

During the past decades, various indirect left-turn designs have been used on urban or suburban multilane arterials to eliminate problems associated with direct left-turn movements at intersections. Using non-traversable medians and/or directional median openings, direct left-turn movements from collector streets or local streets are prohibited. Left-turning vehicles will be redirected to a preselected downstream U-turn location to make U-turns. The median U-turn intersections (MUTI), superstreet intersections, crossover displaced left-turn intersections, the upstream signalized crossover schemes are common U-turn designs [11–15].

**Fig 1:**
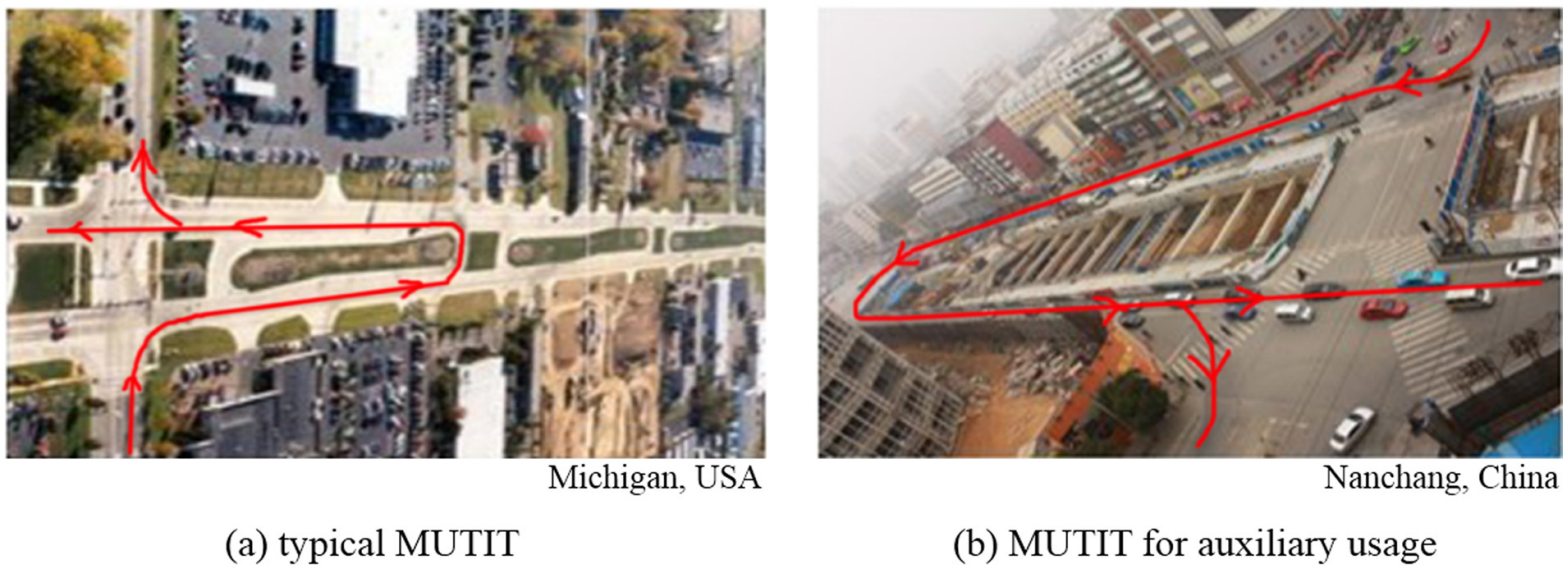
Example of MUTIT application.

The median U-turn intersection treatment (MUTIT), also known as the Michigan U-turn, is the most common design for U-turn vehicles. The geometric configuration of the MUTIT is shown in Fig 1. The design follows three general principles: (1) separate or reduce the conflicts between left-turn movements and the opposing through traffic; (2) reduce the signal phase (e.g. protected left-turn phase); and (3) guarantee the efficiency of other movements on the main street. In present, many local transportation agencies exploit MUTIT as alternatives to direct left-turn movements [16]. Previous studies have reported that the MUTIT can significantly reduce the total delay and improve the overall safety situation at intersection areas [17–20].

The MUTIT has several limitations which restrict its application around the world. One of the limitations is that the MUTIT requires sufficient median width to accommodate the design of U-turn lane. For intersections with narrow median on arterials in both directions, it is very difficult to implement the MUTIT for the U-turn design. In addition, the small vehicles and the large vehicles (i.e., buses, trucks, etc.) have different turning radius which should be considered when designing the U-turn on arterials with narrow median and large volume of heavy vehicles.

**Fig 2:**
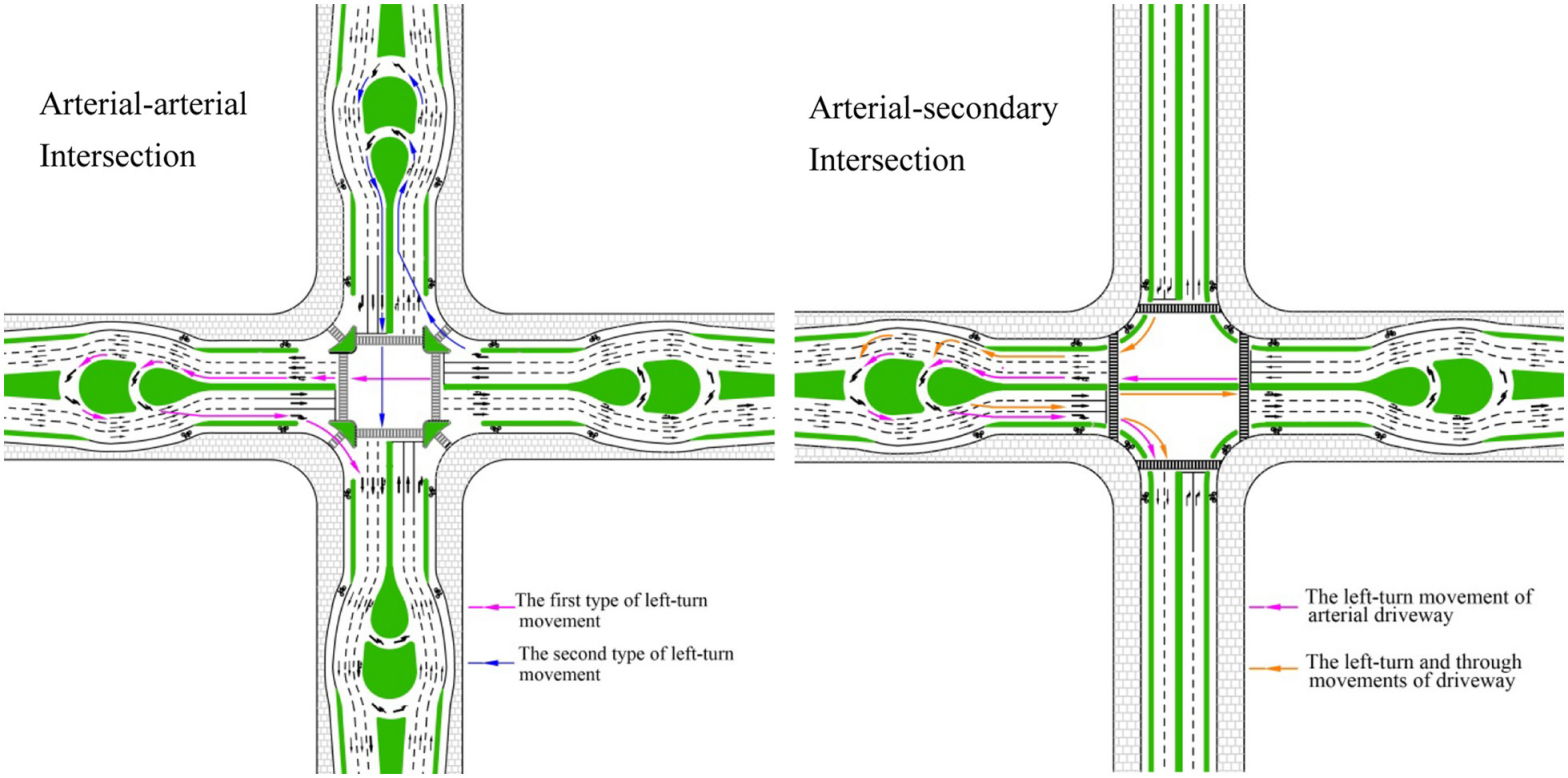
Illustration of UUT at intersection.

In this study, a modified MUTIT design named the unconventional U-turn treatment (UUT) was proposed to relax the unconventional of narrow median. The configuration of the UUT is shown in Fig 2. The left is an arterial-arterial crossing intersection which is usually signalized in order to reduce traffic conflicts, and the right one is an arterial-collector street crossing intersection which often needs no signalization if traffic volume on collector is small. The UUT converts the left-turn movement to be made via two types of median U-turns beyond the intersection. The first one is to travel through the intersection, make a U-turn at the median opening downstream of the intersection, and then turn right at the cross street. The other is to turn right at the intersection and then make a U-turn at the downstream median opening and proceed back through the intersection. The two U-turn path designs are considered beneficial to safety as well because left-turn vehicles have two choices and lane changes can be reduced near intersections.

**Fig 3:**
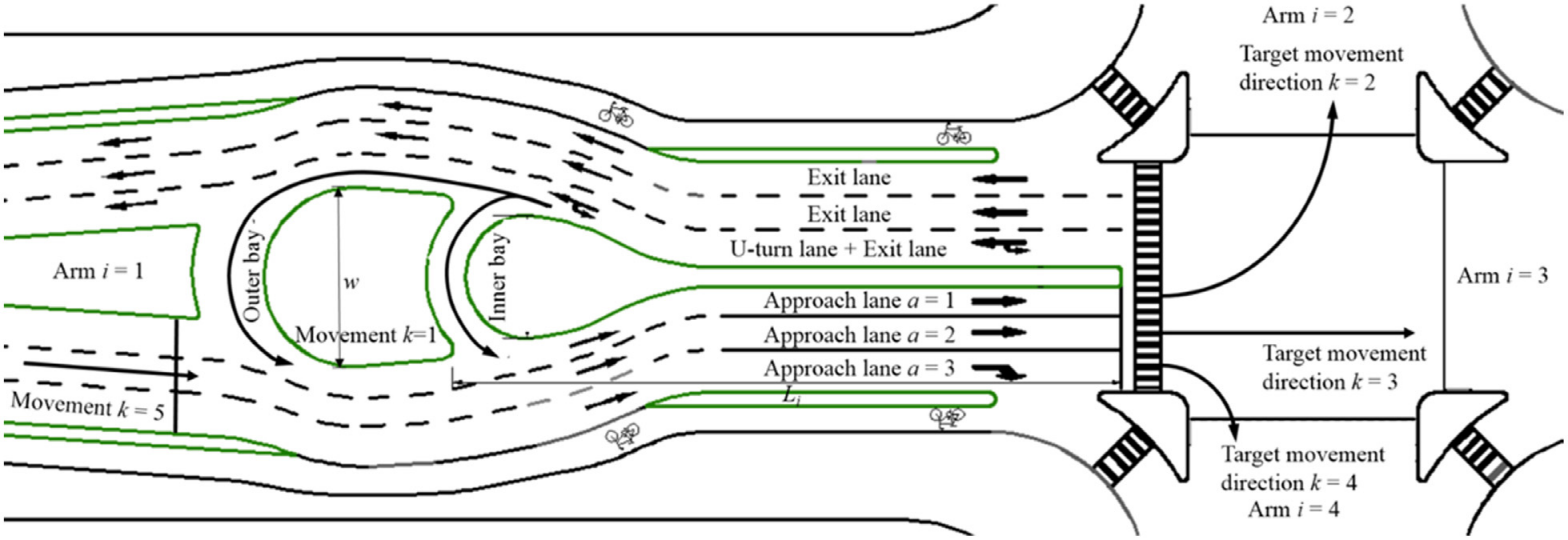
Schematic of UUT in one intersection arm.

In the UUT, the median width of each upstream segment is enlarged only locally in a short distance in order to create an adequate turning radius for the U-turn vehicles. Though some median space is still needed for the UUT design, it is considered much less as compared to that for the MUTIT design. In each intersection arm, the UUT is designed with two U-turn bays: the inner bay (close to the intersection approach) is exclusively prepared for small vehicles, and the outer one is an exclusive U-turn of heavy vehicles, as shown in Fig 3. The dual-bay design is considered helpful to distinguish movements of small and large vehicles and improve the traffic operations. Previous studies have shown that reasonable and coordinated signal control can improve intersection traffic operations [5, 21], and the U-turn signals with the UUT can be designed to cooperatively control with the intersection control for performance improvement.

The primary objective of this study is to evaluate the operational effects of the UUT at intersections. The capacity, delay and number of stops with the UUT were evaluated for various traffic situations. For comparison purpose, the traffic operations with the direct left turn (DLT) for the same intersection were evaluated. In the following section, a review of previous studies is provided. In section 3, the model development is introduced. In section 4, data collection and model calibration are introduced. The simulation results are discussed in section 5. The paper ends with brief concluding remarks and future work in section 6.

## Model Development

### Problem Description

#### Traffic variables

Definitions of traffic variables at UUT and their calculations are introduced in this section. The variables used in the study are summarized in Table 1 and Fig 3.

**Table 1:** Variable definitions used in the model.

Variable	Description
*i*	Index of intersection arms: *i* = 1 for west arm, *i* = 2 for north arm, *i* = 3 for east arm, and *i* = 4 for south arm, as in Fig 3
*k*	Index of turning target movements, *k* = 1 for median U-turn, *k* = 2 for left turn, *k* = 3 for through movement, *k* = 4 for right turn, and *k* = 5 for through movement at median crossover
*a*	Index of approach lanes in one intersection arm, numbered from the left-most lane
*n*_*ia*_	Number of approach lanes in arm *i*
*n*_*ie*_	Number of exit lanes in arm *i*
*L*_*i*_	Distance between median crossover (inner bay) and main intersection in arm *i* (m)
*Q*_*i*_	Demand flow in arm *i* (vph)
*Q*_*ik*_	Demand flow of movement *k* in arm *i* (vph)
*q*_*ia*_	Demand flow of approach lane *a* in arm *i* (vph), *a* = 1,2,3
*t*_*ik*_	The green time of movement *k* in arm *i* (s), *i* = 1,2,3,4
*C*	Signal cycle length (s)
*η*	Clearance time (s)
*w*	Minimum median width for U-turn maneuvers under UUT (m), as in Fig 3
*s*	Minimum spacing of successive vehicles plus the length of one vehicle (m)
*P*(*x*_*j*_)	the probability of a driver *j* selecting the former type
$x_{i}^{\prime }\beta$	the multiple linear combination of explanatory variables (i.e., the utility function)
*ξ*_*i*_	The parameter which is determined as left-turn vehicles divided by total traffic volume (i.e., sum of left-turn, through and right-turn vehicles).

In the study, the green phase of one intersection arm and its opposite arm is assumed to be symmetric. The cycle length *C* is determined by the green phases and the clearance time for both directions, which is:

$$C=\sum_{i=1}^{2}t_{ik}+\eta \begin{array}{cc} & \forall i=1,2\text{ or }i=3,4; \end{array}\;k=2,3,4$$ (1)

The demand flow in arm *i* is the sum of the demand flow of the following three movements:

$$Q_{i}=\sum_{k=2}^{4}Q_{ik}\begin{array}{cc} & \forall i=1,2,3,4 \end{array}$$ (2)

Besides, the demand flow of U-turn movement and through movement at median crossover is identical with the demand flow at intersection approach:

$$\sum_{k=2}^{4}Q_{ik}=Q_{i(k=1)}+Q_{i(k=5)}\begin{array}{cc} & \forall i=1,2,3,4 \end{array}$$ (3)

As stated before, left-turn vehicles can make two types of U-turns at the UUT. The selection of U-turn type where both “straight → U-turn → right-turn” and “turn right → U-turn → straight” roundabouts are determined by the binary logit model [1], which is:

$$P\left(x_{j}\right)=\frac{1}{1+e^{-x_{j}^{\prime }\beta }}\left(j=1,2,\cdots ,J\right)$$ (4)

where *P*(*x*_*j*_) denotes the probability of a driver *j* selecting the former type; $x_{i}^{\prime }\beta$ is the multiple linear combination of explanatory variables which is also known as the utility function. The utility function can be expressed as:

$$x_{i}^{\prime }\beta =\beta_{0}+\beta_{1}x_{1i}+\cdots +\beta_{k}x_{ki}$$ (5)

Consequently, the demand flow of each approach lane in arm *i* is as follows:

$$q_{i(a=1)}=Q_{i2}\cdot \frac{1}{1+e^{\frac{\sum_{j=1}^{J}p\left(x_{j}\right)}{J}}}+\xi_{i}\cdot Q_{i3}\;\begin{array}{cc} & \forall i=1,2,3,4 \end{array}$$ (6a)

$$q_{i(a=2)}=\left(1-\xi_{i}\right)\cdot Q_{i3}\;\begin{array}{ccc} & & \; \end{array}\begin{array}{cc} & \forall i=1,2,3,4 \end{array}$$ (6b)

$$q_{i(a=3)}=Q_{i2}\cdot \frac{1}{1+e^{\frac{\sum_{j=1}^{J}p\left(x_{j}\right)}{J}}}+Q_{i4}\;\begin{array}{cc} & \forall i=1,2,3,4 \end{array}$$ (6c)

where *ξ*_*i*_ is determined as left-turn vehicles divided by total traffic volume (i.e., sum of left-turn, through and right-turn vehicles), which is calculated by

$$\xi_{i}=\frac{Q_{i2}}{\sum_{k=2}^{3}Q_{ik}}\;\forall i=1,2,3,4$$ (7)

#### Geometry

The spacing between median crossover and intersection *L*_*i*_ should be large enough to prevent vehicle spillback at intersections. In this study, *L*_*i*_ should be greater than the vehicle queue length at the intersection approach, which is calculated as:

$$L_{i}\geq \frac{1}{3600}\cdot \frac{Q_{i}}{n_{ia}}\cdot (C-t_{i3})\cdot s\begin{array}{cc} & \forall i=1,2,3,4 \end{array}$$ (8)

In practical applications, the value of *L*_*i*_ could also be determined by the AASHTO Green Book (AASHTO, 2004) which recommends that the minimum spacing between a median crossover and the MUTIT intersection could be between 122m (400ft) and 183m (600ft), if no traffic data are available for calculating *L*_*i*_. Furthermore, the median width for U-turn maneuvers under UUT should be larger than the lower bound value *w*, as shown in Table 2 [7].

**Table 2:** Minimum median widths for U-turn maneuvers under UUT.

Vehicle type	PV	SU	Bus	WB- 50	WB-60
**Vehicle length (m)**	6	9	12	17	21
**Minimum median width *w* (m)**					
**Type of Maneuver**					
** U-turn to left-most lane**	13	23	24	25	25
** U-turn to 2**^***nd***^ **lane**	10	20	21	21	21
** U-turn to 3**^***nd***^ **lane**	7	16	18	18	18

Note: PV, private vehicle; SU, single unit truck; WB-50, semi-truck medium size; WB-60, semi-truck large size.

### Development of Simulation Model

Theoretically, a before—after study in field applications is preferred to compare the operational features of the UUT and DLT design. However, in reality it is very hard to obtain the data before and after the UUT taking place on the same intersection. Thus, simulation technique was used in the study to evaluate the operations of traffic at intersections. Previously, traffic problems have been studied by models such as continuous flow models [22], car following models [23], and microscopic simulation models [15–18]. Among them, the VISSIM simulation model has been considered the most commonly used technique to analyze the traffic flow characteristics at intersections with MUTIT [24–26]. Previous studies have shown that after parameter calibration, the VISSIM model can accurately reflect many important features for U-turn vehicles [18, 27]. Both the traffic situations with and without UUT at an isolated intersection were simulated in our study.

#### Development of VISSIM model

To ensure that the geometric elements of intersections are precisely modeled, the CAD layouts of typical intersections on four-lane and six-lane divided streets were imported into VISSIM. When UUT is executed without U-turn signal control, the priority rule for through and U-turn movements should be defined in VISSIM simulation. In this study, U-turning vehicles must yield to the through traffic which is also confirmed by field observations. U-turn drivers need to wait at the bay area until there is an acceptable headway gap in traffic stream [18]. The gap acceptance behavior is calibrated using actual traffic data.

The turning radius of U-turn vehicles at the bay area can be determined by the median nose width and receiving lane width. Vehicle movements are decided for the simulation model according to field observations. The inner bay is exclusively prepared for small vehicles, and the outer one is an exclusive U-turn of heavy vehicles. In practical applications, the U-turn median width should be large enough to make sure that the vehicle turning speed is consistent to field observations for conventional U-turns, as shown in Table 2.

### Calculation of Operational Measures

Three measures are calculated to evaluate the operational features of the U-turn design, including the delay, number of stops and capacity of intersections. Those measures are commonly used in previous studies for the evaluation of intersection performances [11,28–30].

#### Delay

Delay is measure by the difference of actual travel time and desired travel time without stop. With travel time information, vehicle delay is the combination of average stop delay and travel delay [16]. Average stopped delay is defined as the time spent when vehicle stops at intersection, including the stop time due to signal control and that resulting from vehicle queue. Average travel delay is calculated as the additional travel time increased due to vehicle deceleration, slow moving and acceleration at intersection areas. The delay for UUT and DLT can be shown in the following equations:

$$D_{i}^{U}=d_{i}^{U1}+d_{i}^{U2}\begin{array}{cc} & \forall i=1,2,3,4 \end{array}$$ (9)

where *D*_*i*_^*U*^ denotes the total delay of left-turn vehicles at intersection approach *i* with UUT; *d*_*i*_^*U1*^ denotes the average stopped delay of U-turn vehicles from the entrance lanes to the target exit lanes, and *d*_*i*_^*U2*^ denotes the average travel delay of U-turn vehicles from the entrance lanes to the target exit lanes.

$$D_{i}^{C}=d_{i}^{C1}+d_{i}^{C2}\begin{array}{cc} & \forall i=1,2,3,4 \end{array}$$ (10)

where *D*_*i*_^*C*^ denotes the total delay of left-turn movement vehicles at intersection approach *i* with DLT; *d*_*i*_^*C1*^ denotes the average stopped delay of left-turn vehicles conventional signalized treatment vehicles from the entrance lanes to the target exit lanes, and *d*_*i*_^*C2*^ denotes the average travel delay of left-turn vehicles operating under conventional signalized treatment from the entrance lanes to the target exit lanes. More details on the calculation of the above parameters can be found in [29].

#### Number of stops

The number of stops per vehicle is calculated by the total stop counts divided by the number of vehicles crossing the intersection, which is:

$$S_{i}^{U}=\frac{\sum_{j=1}^{Q_{i2}}s_{ij}^{U}}{Q_{i2}}\begin{array}{cc} & \forall i=1,2,3,4 \end{array}$$ (11)

where *S*_*i*_^*U*^ denotes the average count of stops of left-turn movements at intersection approach *i* with UUT; *s*_*ij*_^*U*^ denotes the count of stops for left-turn vehicle *j* at intersection approach *i* with UUT; *Q*_*i2*_ denotes total number of left-turn vehicles at intersection approach *i* with UUT.

$$S_{i}^{C}=\frac{\sum_{j=1}^{Q_{i2}}s_{ij}^{C}}{Q_{i2}}\begin{array}{cc} & \forall i=1,2,3,4 \end{array}$$ (12)

where *S*_*i*_^*C*^ denotes the average count of stops of left-turn movements at intersection approach *i* with DLT; *s*_*ij*_^*C*^ denotes the count of stops for left-turn vehicle *j* at intersection approach *i* with DLT; *Q*_*i2*_ denotes total number of left-turn vehicles at intersection approach *i* with DLT.

#### Capacity

The capacity is the sum of vehicle count from all movements at intersection area with UUT. For the intersection arm *i*, the capacity is calculated as:

$$C_{i}^{U}=\sum_{k=2}^{4}c_{ik}^{U}\begin{array}{cc} & \forall i=1,2,3,4 \end{array}$$ (13)

where *C*_*i*_^*U*^ denotes the whole capacity at intersection approach arm *i* with UUT; *c*_*ik*_^*U*^ denotes the capacity of vehicles from left-turn, through and right-turn movement respectively. Note that U-turn capacity constitutes the capacity of left-turn movements at approach *i* with UUT.

The whole vehicle capacity at intersection approach arm *i* with DLT is as follows:

$$C_{i}^{C}=\sum_{k=2}^{4}c_{ik}^{C}\begin{array}{cc} & \forall i=1,2,3,4 \end{array}$$ (14)

where *C*_*i*_^*C*^ denotes the whole capacity at intersection approach arm *i* with DLT; *c*_*ik*_^*C*^ denotes the capacity of vehicles from left-turn, through and right-turn movement respectively. The detailed calculation of vehicles capacity of each movement is also referred to [29].

## Data

### Data Collection

Field data collection was conducted to obtain the data for model calibration. There is no specific permission required for these locations. Field data were collected from six intersections in the urban public area of Nanchang, China, other than private lands. Video cameras were used in the data collection at intersections. The field studies did not involve endangered or protected species. The city for data collection has a population of 5.6 million and an area of 7,402 square kilometers by 2013.

Firstly, two intersections were selected for data collection in order to calibrate the simulation model. Because that the UUT has not been implemented in the field, our study selected intersections with geometric features similar to the UUT to calibrate the vehicle maneuver parameters in the VISSIM simulation model. The selected sites are located at intersections of Beijing East Rd—Shanghai North Rd (coordinates: 115.940728, 28.674267), and Beijing East Rd—Qingshanhu Rd (115.948881, 28.674354).

Secondly, four other intersections were selected for data collection during weekdays including both peak and off-peak periods. Those sites were used to evaluate the performance of the UUT compared with the DLT. The geometric features and traffic information at each site are shown in Tables 3 and 4. Intersection 1 and 2, which are the intersection of Hongduzhong Ave—Ruoyang Rd (115.928111, 28.663710), and that of Hongduzhong Ave—Nanjing West Rd (115.926169, 28.684644), are located in central business districts. The other two, which are the intersection of Fenghezhong Ave—Huizhan Rd (115.854629, 28.688569), and Fenghezhong Ave—Lüyin Rd (115.860144, 28.693736) are located in non-central business districts. The peak-hour traffic demands were estimated by traffic volume, as shown in Table 4, which were expanded according to queue length in intersections entrance lanes. The peak-hour demands of four intersections from site1 to site 4 are respectively 2825 pcu/h, 2769 pcu/h, 6140 pcu/h, 8200 pcu/h. The capacities of the intersections were evaluated by channelization, signal scheme, and effects of non-motor vehicles and pedestrian. The actual capacities of four intersections from site1 to site 4 are respectively 5300 pch/h, 5200 pch/h, 5160 pch/h, 6080 pch/h. Please note that before the UUT design is fully evaluated by researchers, it cannot be implemented in the field. Thus, the study cannot obtain actual traffic data from the intersections with UUT. In our study, the performance of UUT was evaluated in the VISSIM simulation model which was calibrated using data collected in the field. In the VISSIM simulation model, the distance between median crossover (inner bay) and main intersection is set to be 130 m according to Eq (8). The median widths of inner bay and outer bay are set to be 10 m and 18 m respectively, according to Table 2.

**Table 3:** Basic geometric feature of selected intersections.

Sites	Type	Number of approach lane (NS)			Number of approach lane (WE)		
		left turn	straight	right turn	left turn	straight	right turn
**1**	four-leg	2	3	1	1	2	1
**2**	four-leg	2	3	1	1	2	1
**3**	four-leg	2	3	1	1	2	1
**4**	four-leg	2	3	1	1	3	1

**Table 4:** Traffic volume conditions of selected intersections.

Sites	Volume of northbound (pcu/h)			Volume of southbound (pcu/h)			Volume of eastbound (pcu/h)			Volume of westbound (pcu/h)		
	left turn	straight	right turn	Left turn	straight	right turn	left turn	straight	right turn	left turn	straight	right turn
**1**	100	397	167	182	432	282	117	345	197	138	317	151
**2**	91	345	96	254	381	127	332	272	127	181	454	109
**3**	200	1200	500	180	1500	500	170	700	140	190	560	300
**4**	550	1500	500	300	1200	200	200	1000	450	550	1550	200

### Calibration of Simulation Model

The VISSIM model was calibrated and validated against field data to ensure that it simulates accurate traffic operation. To calibrate the selected parameters in the simulation model, field data were collected including traffic data, geometric features, and drivers’ behavioral information.

Initially, the logit model for predicting driver’s choice of two U-turns was calibrated. A total of 316 U-turn behaviors were identified in the field observation. The percentages of the two U-turns are 52.6% and 47.4% respectively. The green signal for the through and right-turn movement was 40 s and 30 s respectively.

Some data gathered in the field were considered as the inputs in the VISSIM: 1) The percentage of private vehicles and heavy vehicles are 96% and 4% respectively; 2) The headway of private vehicles ranges from 4.0 s to 14.0 s which an average of 6.9 s; 3) The headway of heavy vehicles ranges from 7.4 s to 11.0 s with an average of 9.8 s; 4) The turning speed of private vehicles is 12.3 km/h, and the speed ranges from 7.1 km/h to 18.7 km/h; and 5) The turning speed of heavy vehicles is 9.7 km/h and the speed ranges from 7.2 km/h to 10.8 km/h.

Several parameters in the VISSIM model needs to be calibrated which are the parameters in the gap-accepting model, the car-following model, and the lane-changing model. Considering that the capacity is one of the most important factors in the evaluation of U-turn movements, the capacity was considered as the measurement of goodness of fit to calibrate the predictions in the VISSIM model. This study used the Kyte’s method [31] to measure U-turn capacity in the field.

The method proposed by Kyte in 1991 was to measure field capacity in an under-saturated condition, which is described in the following equation:

$$c_{f}=\frac{3600}{t_{s}+t_{mv}}$$ (15)

where *c*_*f*_ denotes the capacity of U-turn movement measured in the field (veh/h); *t*_*s*_ denotes the average service delay for each U-turning vehicle (s), which is defined as delay occurs at the first position of the U-turn queue; and *t*_*mv*_ denotes average move-up time for each U-turning vehicle (s), which is the amount of time from when the previous U-turning vehicle exits the stop line until the subsequent queued vehicle reaches the stop line.

Besides, capacity was selected as the goodness of fit measure for validation of the calibrated simulation model. The index used for measuring simulation error is the mean absolute percent error (MAPE), which can be estimated as:

$$MAPE=\frac{1}{n}\sum_{i=1}^{n}\left|\frac{c_{s}^{i}-c_{f}^{i}}{c_{f}^{i}}\right|$$ (16)

where *n* denotes the number of video sections, *c*_*s*_^*i*^ is the capacity of U-turn movement estimated by the VISSIM simulation model (veh/h), and *c*_*f*_^*i*^ is the field capacity of U-turn movement at time interval (veh/h).

Table 5 presents the model validation results. Results show that VISSIM simulation models yield a MAPE of 19.7% and 23.3% for private vehicles and heavy vehicles respectively. The estimation error can be considered acceptable in practical engineering applications.

**Table 5:** Model validation results.

Vehicle type	Small vehicles		Heavy vehicles	
Intersection site	B.E. Rd.-S. N. Rd	B.E. Rd.-Q. Rd.	B.E. Rd.-S. N. Rd	B.E. Rd.-Q. Rd.
**Simulated capacity (veh/h)**	646	605	377	351
**Measured capacity (veh/h)**	541	504	312	279
***Model fitness***				
**MAPE (%)**	19.7%		23.3%	

## Results

### Operational Features with UUT and DLT Design

The VISSIM simulation models are developed for the same intersections to evaluate the traffic operational features, including capacity, delay, and number of stops. Actual traffic parameters such as flow and traffic components collected in the field are inputted in the simulation model.

The simulation results for four intersections with both UUT and DLT design, according to traffic demands in Table 4, are summarized in Table 6. The UUT is designed without an exclusive U-turn signal phase. Table 6 shows that the UUT design increased the intersection’s system capacity by 5.10% to11.20%, decreased the vehicle delay by 2.1% to 40.1%, and cut down the average number of stops by 0.35% to 36.62%, as compared to the DLT. The UUT design effectively improved the performances of site 1 and 2, where traffic demands were less than 3000 pcu/h and the volume/capacity ratio was 0.48 and 0.50. However, the performances of site 3 and site 4 were improved very little with the UUT design, where traffic demands were very high and the volume/capacity ratio was 1.07 and 1.28. Furthermore, it is found that the performance of site 1 was better than that of site 2, while the volume/capacity ratios were very close. The ratio of left-turn to through traffic volume at the two intersections was 0.36:1 and 0.59:1 respectively. Similarly, the performance of site 3 was better than site 4 when the volume/capacity ratio of site 3 was close to that of site 4, and the ratio of left-turn to through traffic volume was 0.19:1 and 0.30:1 respectively.

**Table 6:** Performance of UUT and DLT based on field traffic volume.

Sites	Traffic demand	Capacity (pcu/h)			Delay (s)			Average number of stops		
		DLT	UUT	Rate ^a^ (%)	DLT	UUT	Rate ^a^ (%)	DLT	UUT	Rate^a^ (%)
**1**	2825	5300	5850	10.38	39.20	23.48	-40.1	0.71	0.45	-36.62
**2**	2769	5200	5710	9.81	42.70	34.79	-18.5	0.73	0.56	-23.19
**3**	6140	5166	5745	11.20	249.30	237.83	-4.60	2.67	2.62	-1.87
**4**	8200	6080	6390	5.10	260.10	254.64	-2.10	2.89	2.88	-0.35

^*a*^ *Rate* = (UUT-DLT)/DLT∙100%.

The above results show that the UUT design has the better potential to effectively improve the operational performance at intersections than the DLT design. The UUT is more suitable for designing intersection with small the value of ratio between volume and capacity and low ratio of left-turn to through traffic volume.

### Sensitivity Analysis of Operational Performance

Field data collection does not cover all possible traffic situations which restricts the full evaluation of operational effects of the UUT design. In this section, different traffic situations are specified in the VISSIM simulation model to further investigate the differences between the UUT and DLT design. The intersection used for simulation is crossed by an arterial (6 legs) and a collector (4 legs) and the whole capacity for the intersection is 5800 pcu/h. At first, sensitivity analysis of delay and number of stops are calculated for different traffic situations. The traffic volume on the arterial street is specified to range from 600 to 2400 pcu/h, with an increase of 600 pcu/h. The traffic volume on the collector increases from 100 to 1500 pcu/h, with an increase of 200 pcu/h.

**Fig 4:**
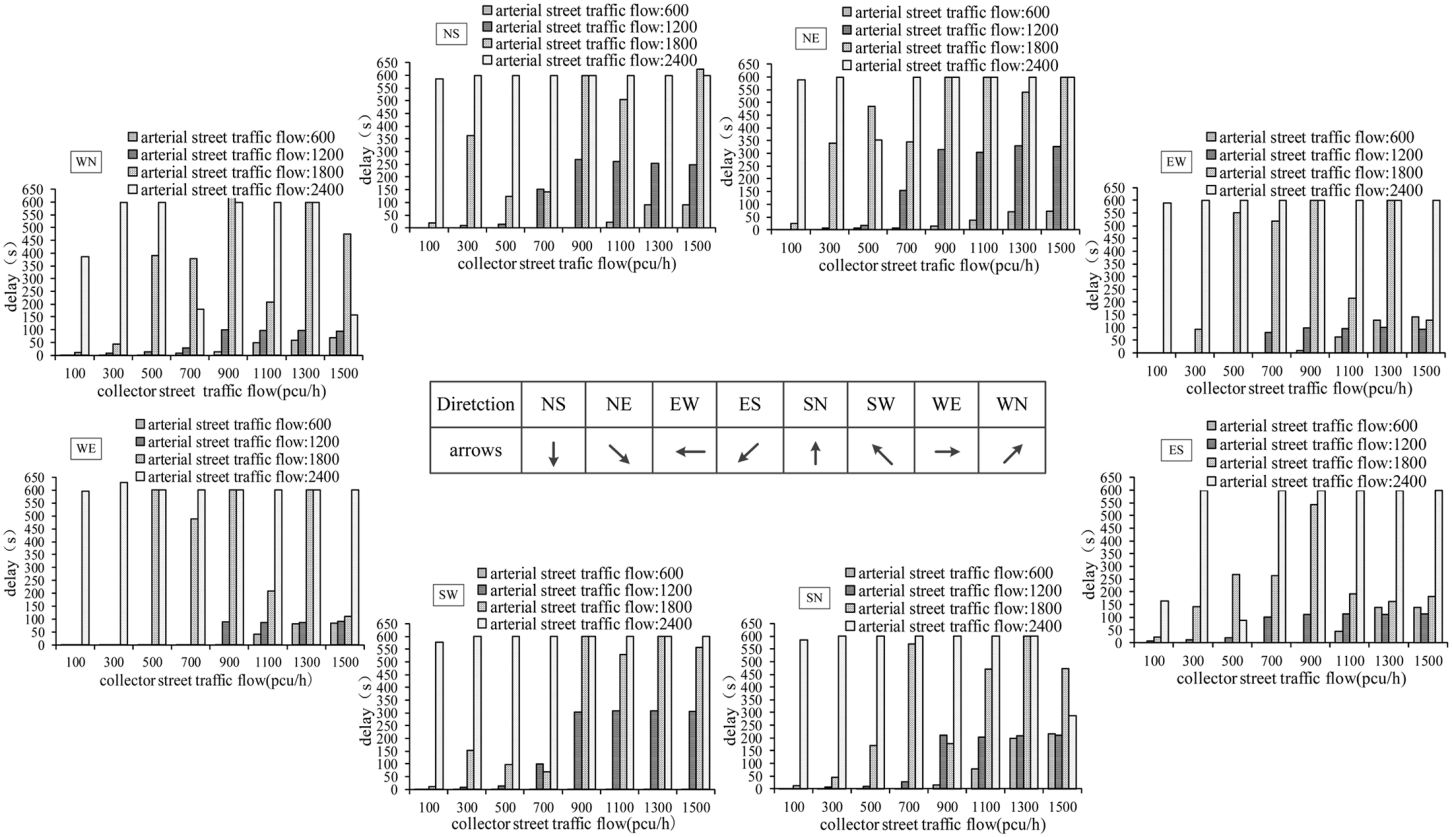
Vehicle delay for through and left-turn movements.

Vehicle delay under different traffic situations are shown in Fig 4. When volume on arterial street is 600, 1200, 1800 pcu/h and volume on collector street is less than 1100, 500, 100 pcu/h, average vehicle delay is very small (i.e., no more than 15 s). When volume on arterial or collector street continuously increases, the vehicle delay will dramatically increase and the intersection becomes very congested. For example, when volume on arterial street is 1200 pcu/h and on collector street is 900 pcu/h, the vehicle delays will be over 93 s. Similar result occurs when arterial street volume is 1800 pcu/h and collector street volume is 300 pcu/h. when arterial street volume is 1800 pcu/h and collector street volume is 500 pcu/h, the vehicle delay will be over 2 minutes. When arterial street volume is 1800 pcu/h and collector street volume is 700 pcu/h, the vehicle delay will be close to 5 minutes, which will finally result in the occurrence of the spillback congestion.

**Fig 5:**
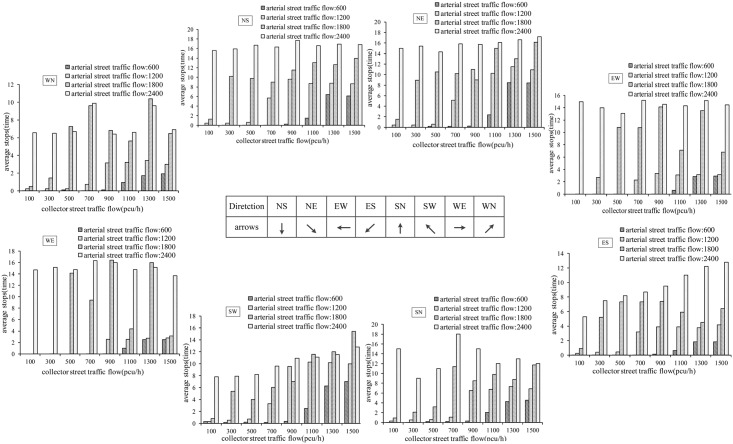
Average count of stops for through and left-turn movements.

The number of stops is also analyzed for different traffic situations. The results are shown in Fig 5. When arterial street volume is 600, 1200, 1800 pcu/h respectively and collector street volume is less than 1100, 500, 100 pcu/h, the number of stops is considered acceptable (no more than 1.5). However, as arterial or collector street volume increases, the number of stops for each vehicle significantly increases. For example, when arterial street volume is 1200 pcu/h and collector street volume is 700 pcu/h, the number of stops is more than 5. When collector street volume is over 900 pch/h, the number of stops is more than 9 and the intersection is in serious congestion. Similar result occurs when arterial street volume is 1800 pcu/h and collector street volume is more than 500 pcu/h.

In summary, according to the results from sensitivity analyses, it is can be found that the intersection operates fluently if traffic demand is less than 1700 pcu/h. Furthermore, when the volume/capacity ratio of the intersections is less than 0.58, the UUT is a good design.

**Fig 6:**
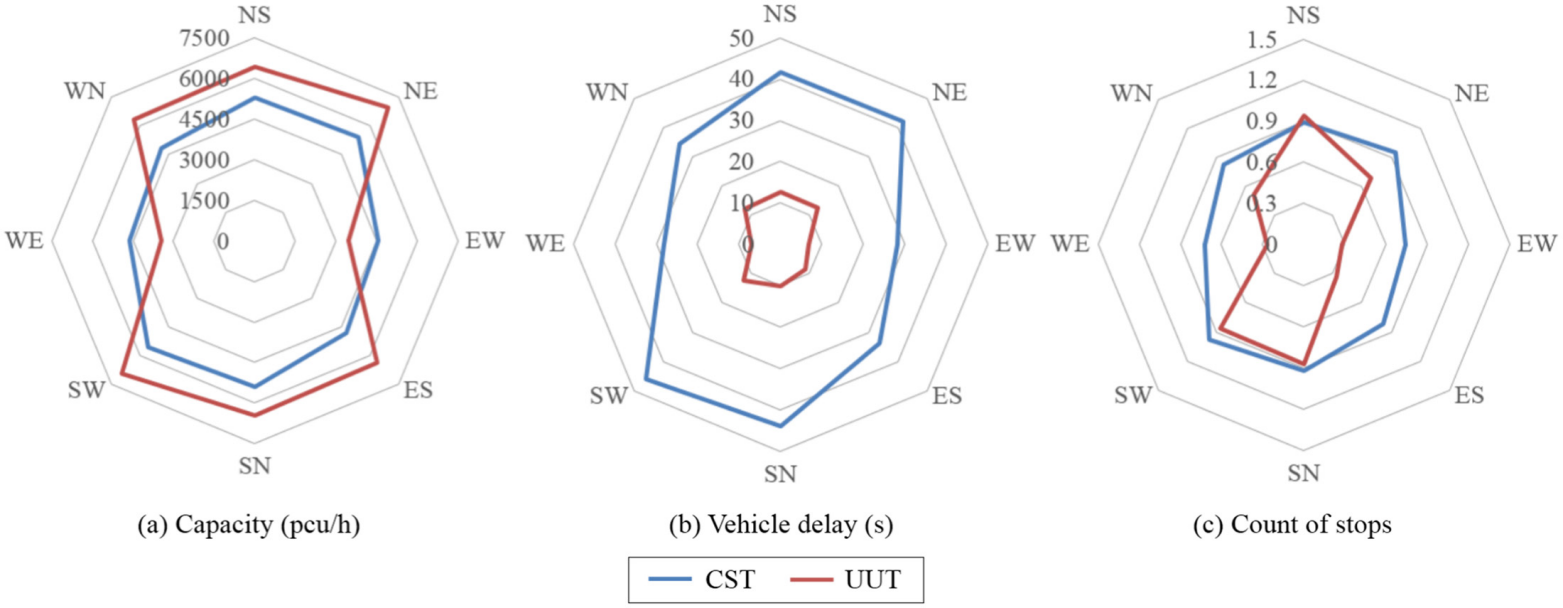
Performance of UUT and DLT under high through volume and moderate left-turn volume.

A comparison of operational effect between UUT and DLT was also conducted. When the intersection operates with the ratio of left-turn to through traffic volume less than 0.33:1, UUT has better performance than DLT in terms of higher capacity, less delay and number of stops. Fig 6 presents the comparison between UUT and DLT, given that the arterial street volume is 1200 pcu/h, the collector street volume is 500 pcu/h, and the ratio of left-turn to through traffic volume is 0.33:1. The vehicle delay in eight directions is significantly lower in the case of UUT than that of DLT. Though in some cases the capacity and number of stops for DLT is better than UUT, the overall operational performance for UUT is considered better than the DLT.

### Advanced Signal Control with UUT

The sensitivity analysis shows that when the ratio of left-turn to through traffic volume is close to or larger than 1, the advantage of the UUT design is not quite obvious as compared to the DLT. The reason for this is that as the increase of flow and decrease of headway, traffic conflicts are more severe because: (1) the U-turn vehicles cannot merge into the mainstream traffic stream easily; and (2) the right-turn vehicles cannot merge into the mainstream traffic stream easily. To further improve the operational performance, two advanced signal control strategies in conjunction with UUT are proposed.

**Fig 7:**
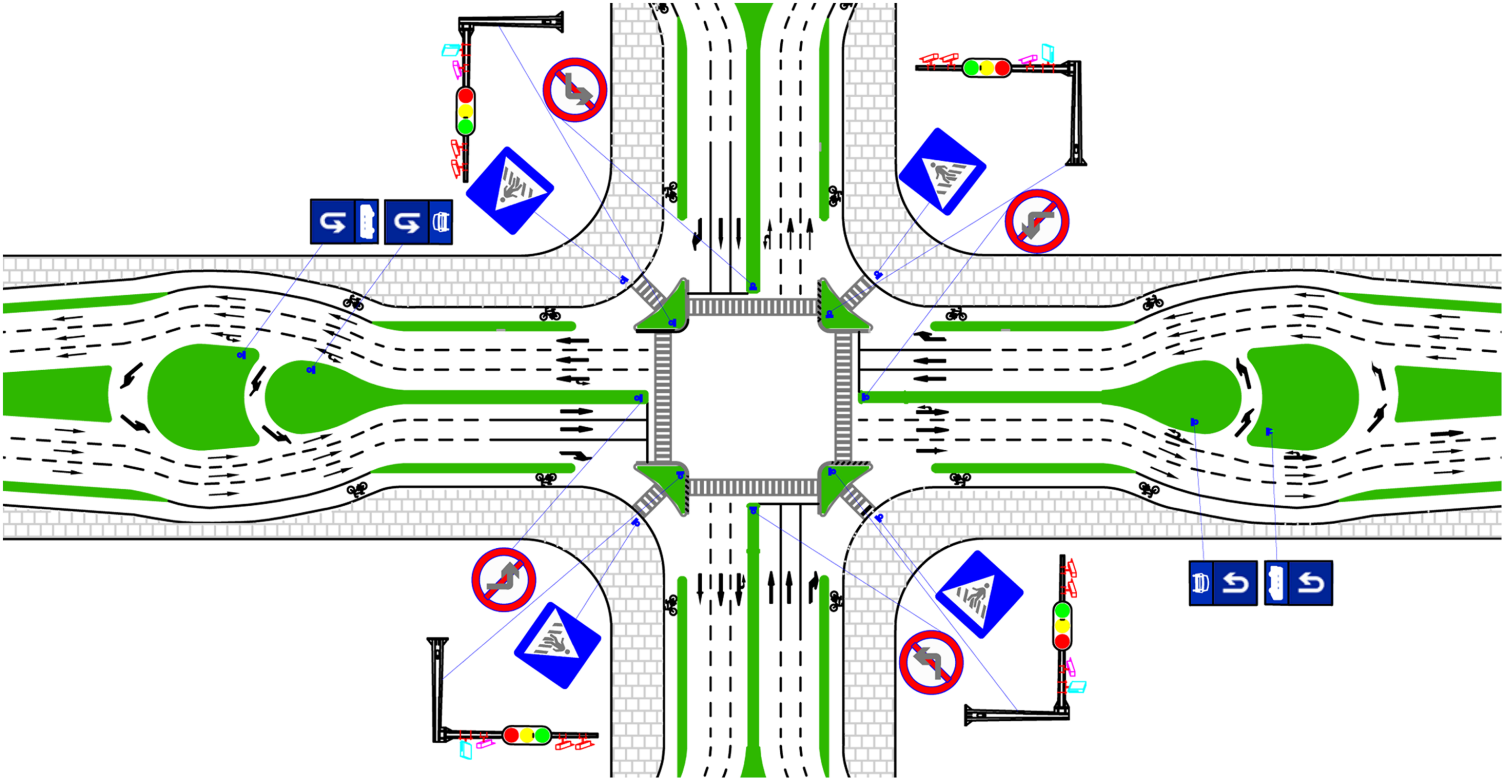
First strategy: the UUT plus signalized control at the intersection approach.

The first strategy is the UUT plus signalized control at the intersection approach (denotes as UUT plus A), as shown in Fig 7. In this strategy, the right turn was protected by signal so that the conflicts could be resolved between right-turn U-turn vehicles and mainline traffic stream.

**Fig 8:**
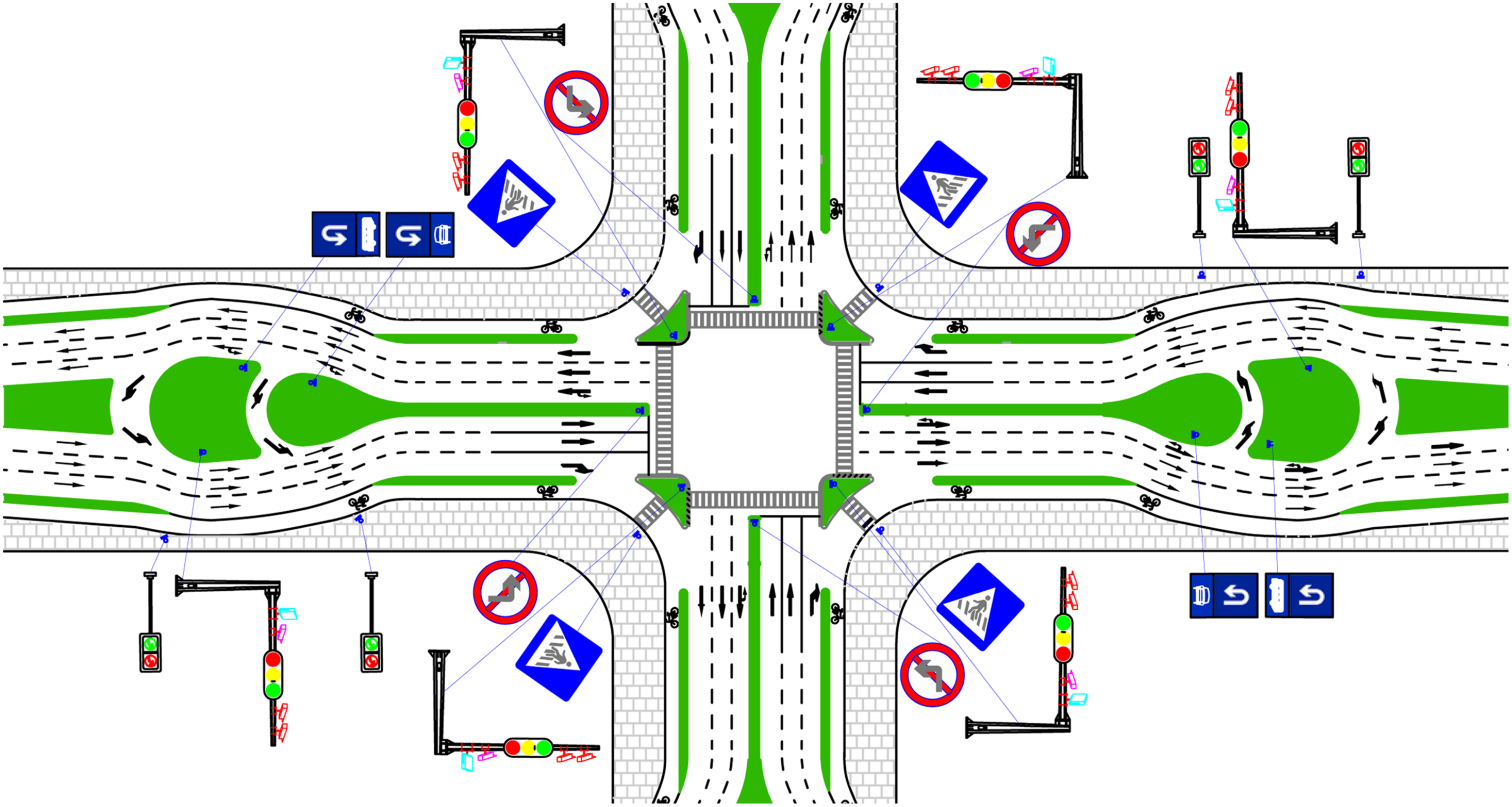
Second strategy: the UUT integrated signalized control at both the U-turn bay and intersection approach.

**Fig 9:**
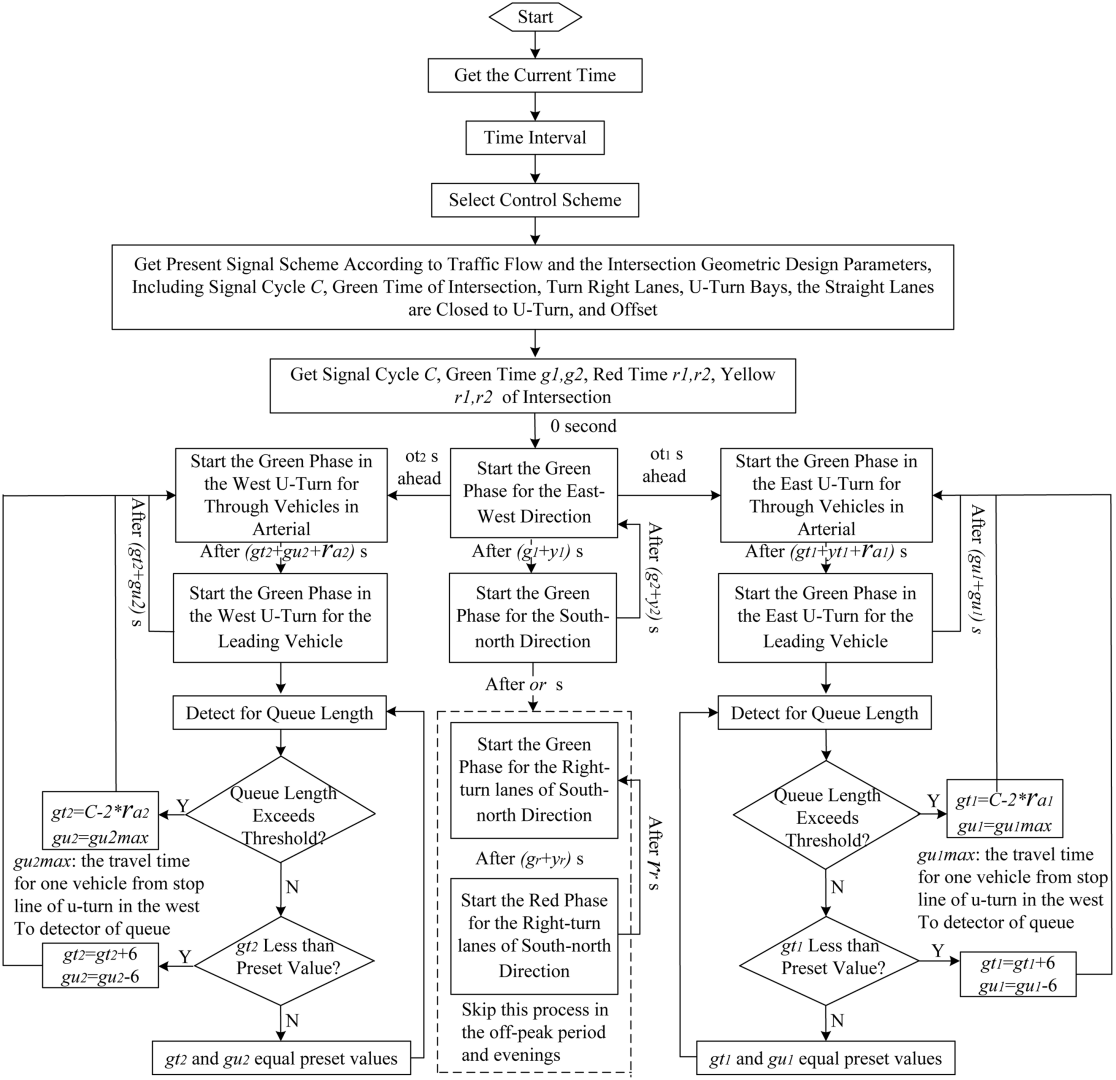
Signal control logic in the second strategy.

The second strategy is to use the UUT pluses an integrated signalized control at both the U-turn bay and the intersection approach (denotes as UUT plus B), as show in Fig 8. This strategy uses adaptive signal control to adjust the signal timing according to real-time traffic situations, in order to improve the efficiency of signal and to reduce the impact of queuing vehicle spill over. The control logic is shown in Fig 9.

**Fig 10:**
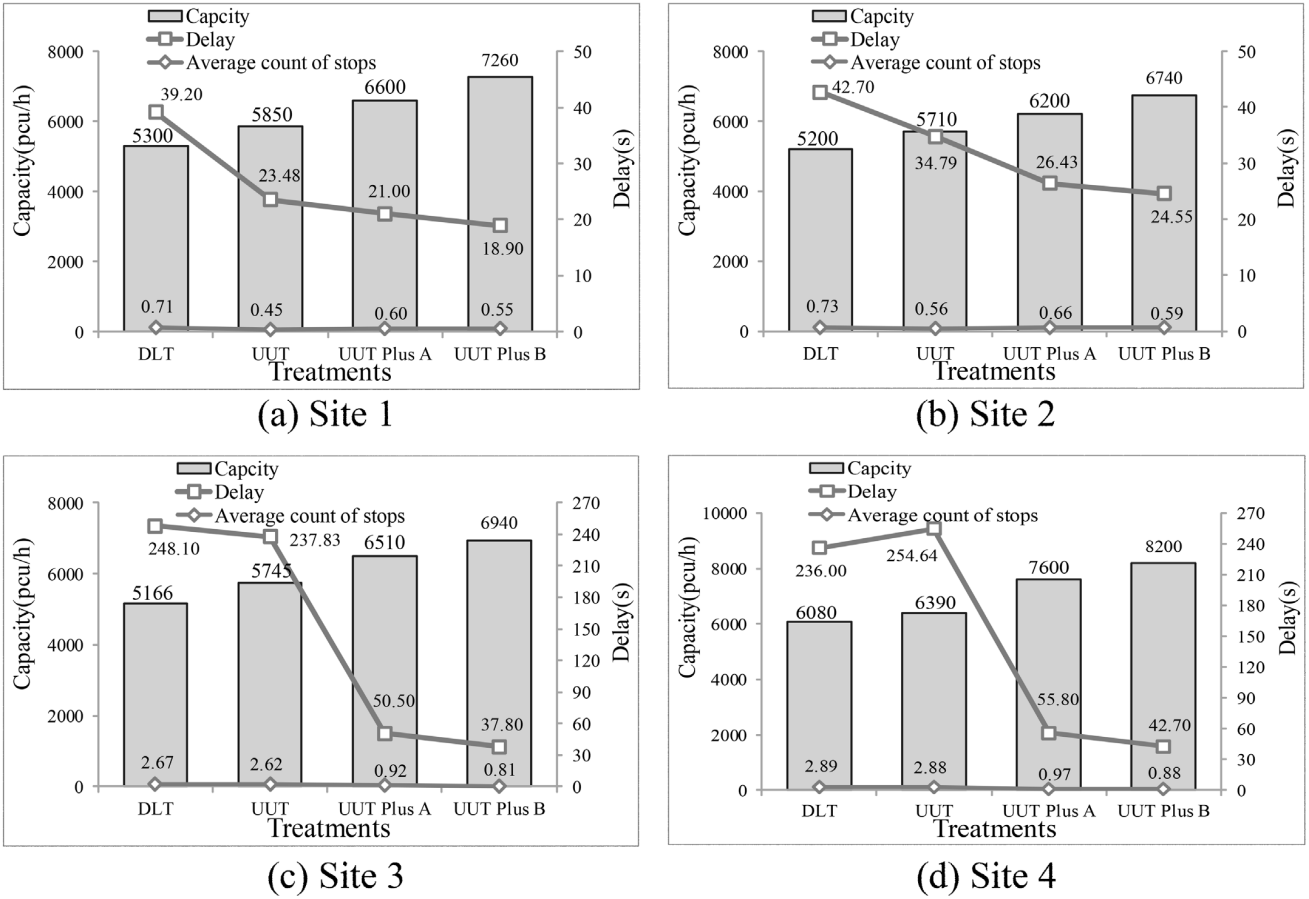
Performance of four treatments based on field traffic volume.

Four intersections, as shown in Table 3, were considered in the simulation analysis according to traffic demands in Table 4. The results of the second signal control strategy with UUT are shown in Fig 10.

UUT plus A can effectively improve the capacity of intersections and reduce the delay when compared with UUT only, especially for the intersections of large volume/capacity ratio. Capacities of intersections from site1 to 4 are increased by 13%, 9%, 13% and 19%, and delay of each site is decreased by 11%, 24%, 79% and 78%, respectively. However, the average number of stops at site1 and 2 is increased by 34% and 17% respectively, where the volume/capacity ratio was small. Actually, the average number of stops of left-turn vehicles increased due to signal control. Meanwhile, the average number of stops of site 3 and 4 significantly decreased where the volume/capacity ratio was large. In addition, the design of UUT plus B further increased the capacity and reduced the delay and number of stops at intersection areas. For example, with the UUT plus B, the capacity is increased by more than 8%, the delay is decreased by over 7%, and the average number of stops is decreased by more than 9% for the same intersections.

The results showed that the UUT design with integrated signal control can effectively increase the capacity and reduce the delay and vehicle stops at intersections, as compared with either UUT only or DLT. The UUT plus B has the best operational performance. In addition, the simulation results also showed that with the integrated signal control, the UUT can still effectively improve intersection operations in the traffic situations with large volume/capacity ratio.

## Conclusions

Traditional MUTIT has the limitation in the requirement of large median width to accommodate the U-turn lane. In this study, a new U-turn design, named the unconventional U-turn treatment (UUT) is proposed. The core design of UUT is that the median is enlarged only locally to accommodate larger turning radius for U-turn vehicles but doesn’t occupy large median space. The UUT is designed with two U-turn bays with different radiuses for small vehicles and large vehicles respectively. The VISSIM simulation model is developed and calibrated to evaluate the operational features at intersections with UUT. Traditional DLT design is also evaluated for comparison.

The results show that when the intersection operates with small volume/capacity ratio and low ratio of left-turn to through traffic volume, UUT has an obviously better performance than the DLT. In the case, the capacity is increased by 9.81% to 10.38%, vehicle delay is decreased by 18.5% to 40.1%, and number of stops is decreased by 23.19% to 36.62% when the volume/capacity ratio is less than 0.50. When the traffic volume of through and left-turn traffic is large, UUT has very little improvement as compared to DLT, but UUT with signal control (UUT plus A) presents obvious advantage over UUT only. Compared with DLT, the capacity is increased by 25% to 26.02%, vehicle delay is decreased by 50.5% to 55.8%, and number of stops is decreased down to 69.5% when the volume/capacity ratio is more than 1. Furthermore, the UUT in conjunction with signal control at both U-turn bay and intersection approach (UUT plus B) is the most appropriate measure to cope with high left-turn and through traffic volume, because it effectively reduced delay and number of stops.

The findings of this study can provide useful information in understanding the applicability of UUT under different traffic conditions. It can be utilized as a guideline for transport policy makers and planners to determine when and where the UUT should be used. Before the UUT being used in practical applications, some issues can be further studied in the future. First, the distance between two U-turn bays in one intersection arm is fixed in this study, which can be relaxed to consider its effect on UUT. Second, this study only investigates an isolate intersection. The study scope could be extended to a broader spectrum for simulating a larger network to more precisely estimate the operational effects of UUT. In addition, the performance of DLT could be further improved by optimization of signal control. It would be interesting to compare the UUT and DLT design when both are integrated with advanced signal control techniques. The authors recommend that future studies could focus on these issues.
