# Correction: Evaluating the Operational Features of an Unconventional Dual-Bay U-Turn Design for Intersections

**DOI:** 10.1371/journal.pone.0163758

**Published:** 2016-09-22

**Authors:** The PLOS ONE Staff

There are errors in the funding section. The correct funding information is as follows: This work was jointly supported by the Key Program of National Natural Science Foundation of China (No. 51338003), the Science and Technology Cooperation and Achievement Transformation Achievement Transformation Program (No. 2013HZCG023), the National Science Foundation of China (No. 51478113, No. 51508094) and of Jiangsu Province (BK20150612), the Fundamental Research Funds for the Central Universities, the Postgraduate Research and Innovation Plan Project of Jiangsu Province (No.KYLX15_0154), and the Key research and development project of Jiangxi Province (20161BBG70044). The funders had no role in study design, data collection and analysis, decision to publish, or preparation of the manuscript. The publisher apologizes for the errors.
